# Assessment of aflatoxin B1 in livestock feed and feed ingredients by high-performance thin layer chromatography

**DOI:** 10.14202/vetworld.2015.1396-1399

**Published:** 2015-12-17

**Authors:** Korrapati Kotinagu, T. Mohanamba, L. Rathna Kumari

**Affiliations:** Toxicology and Feed Analysis Laboratory Veterinary Biological Research Institute, Shanti Nagar, Hyderabad, Telangana, India

**Keywords:** aflatoxin B1, animal feed, feed ingredients, high-performance thin layer chromatography

## Abstract

**Aim:**

Detection of aflatoxin B1 in Livestock compound Feed and feed ingredients by high-performance thin layer chromatography (HPTLC).

**Materials and Methods:**

Chromatography was performed on HPTLC silica gel 60 F 254, aluminum sheets by CAMAG automatic TLC sampler 4, with mobile phase condition chloroform:acetone:water (28:4:0.06). Extraction of aflatoxin B1 from samples was done as per AOAC method and screening and quantification done by HPTLC Scanner 4 under wavelength 366 nm.

**Results:**

A total of 97 livestock feed (48) and feed ingredients (49) samples received from different livestock farms and farmers were analyzed for aflatoxin B1of which 29 samples were contaminated, constituting 30%. Out of 48 livestock compound feed samples, aflatoxin B1 could be detected in 16 samples representing 33%, whereas in livestock feed ingredients out of 49 samples, 13 found positive for aflatoxin B1 representing 24.5%.

**Conclusion:**

HPTLC assures good recovery, precision, and linearity in the quantitative determination of aflatoxin B1 extracted from Livestock compound feed and feed ingredients. As more number of feed and feed ingredients are contaminated with aflatoxin B1 which causes deleterious effects in both animal and human beings, so there is a need for identifying the source of contamination, executing control measures, enabling better risk assessment techniques, and providing economic benefits.

## Introduction

Mycotoxins are structurally fungal metabolites produced by Fungi, not essential to fungal growth and produced periodically under fungal stress. They can contaminate a variety of mixed feed and food leading to animal and human health specific component species such as *Aspergillus*, *Fusarium*, and *Penicillium* are able to produce these toxins that account per annum for millions of dollars in losses worldwide in condemned agriculture products [1].

Fungal metabolites generally associated with fungi belonging to the genera *Alternaria*, *Aspergillus*, *Fusarium*, and *Penicillium*. Toxigenic *Alternaria* and *Fusarium* species are often classified as field fungi, while *Aspergillus* and *Penicillium* species are considered storage fungi. The most common *Fusarium* mycotoxins are trichothecenes, zearalenone, and fumonisins. The mycotoxins of interest produced by *Aspergillus* species include aflatoxin and ochratoxin A, while *Penicillium* species produces ochratoxin A, citrinin and patalin among the more important mycotoxins [2].

Among the mycotoxins that are known to cause human diseases, aflatoxin has been studied most [3]. Aflatoxin was discovered some 30 years ago in England following a poisoning outbreak causing 100,000 turkeys death. Fungi produce aflatoxin in the presence of higher moisture, temperature, and adequate substratum. Before harvest, the risk for the development of aflatoxin is greatest during major droughts, where soil moisture is below normal and temperatures are high, the number of *Aspergillus* spores in the air increases. These spores infect crops through areas of damage caused by insects, and inclement weather. Once injected, plant stress occurs; the production of aflatoxin is favored. During postharvest stage, proliferation of aflatoxin can be exacerbated in susceptible environment [4]. Today aflatoxin has been one of the most important global concerns regarding contamination of food products [5].

Taking into account the development in *Codex alimentarius*, recently EC has introduced the maximum accepted/residue levels for aflatoxin inanimal feeds as 0.02 mg/kg, i.e., 20 ppb in all feed materials and in the most complete and complementary feedstuffs for cattle, sheep, goats, pigs and poultry, while it is 0.005 mg/kg in complete feeding stuffs for dairy animals and 0.01 mg/kg for complete feeding stuffs for calves and lambs [6].

Thin layer chromatography (TLC) techniques were extensively used for aflatoxin analysis, although recently an increase in the use of High-Performance TLC (HPTLC) has been noted. The accuracy of TLC is less than that of HPTLC, but the results obtained using HPTLC are similar to that of HPLC and a more consistent than Enzyme-linked immunosorbent assay data [7]. In the present study, we have analyzed the feed samples and feed ingredients by HPTLC for aflatoxin B1 contamination.

## Materials and Methods

### Ethical approval

There is no need of ethical approval for such type of study.

### Collection of samples

The livestock feeds and feed ingredients received at toxicology and feed analytical laboratory, Veterinary Biological Research Institute, Hyderabad during the year 2014-2015 were utilized for the present study, A total of 97 livestock compound feeds and livestock feed ingredients received from all over the state of Andhra Pradesh and Telanaga were analyzed for the detection of aflatoxin B1.

### Method validation

All the reagents were of analytical grade (HPLC). The mycotoxins standards, aflatoxin B1 were obtained from Sigma-Aldrich laborchemikalien. The standards were calibrated and checked for its purity by UV spectrophotometer (AOAC, 2000). The Mycotoxins standards solution used in the present study were dissolved in specific solvent (methanol). The extraction of sample were done as per AOAC method and quantified with reference standards by HPTLC.

**Figure-1:**
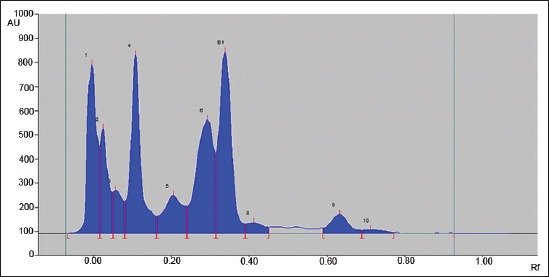
Elution pattern of aflatoxin B1 in feed sample at 366 nm wavelength by CAMAG high-performance thin layer chromatography scanner.

**Figure-2:**
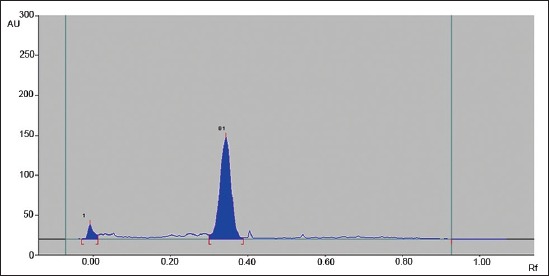
Elution pattern of aflatoxin B1 standard at 366 nm wavelength by CAMAG high-performance thin layer chromatography scanner.

**Figure-3:**
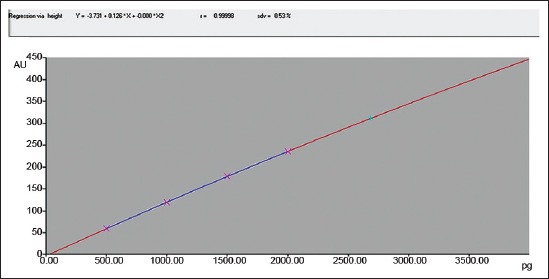
Calibration curve of aflatoxin B1 standard at 366 nm wavelength by CAMAG high-performance thin layer chromatography scanner.

Silica gel 60 F 254 (Merk, Darmstadt) HPTLC plates in the format of 10 × 10 or 20 × 10 were used for analysis. The samples after extraction and drying redissolved with 1 ml of chloroform were applied as bands (spray-on Technique) using CAMAG Automatic TLC sampler 4. The spotted plates were developed in presaturated Twin-trough chamber up to 80 mm from lower edge of the plate with Chloroform:Acetone: water (28:4:0.06) as a mobile phase. After development, the plates were dried using hair dryer and finally the plates were scanned in CAMAG HPTLC Scanner 4 under 366 nm wavelengths to determine the levels of aflatoxin B1 contamination in samples (Figure-1). The standard solutions were applied as the upper method, representing 0.5 ng, 1 ng, 1.5 ng, and 2 ng aflatoxin B1 (Figure-2). The calibration curve of peak area against concentration was plotted (Figure-3). The limit of detection of aflatoxin B1 was 0.02 ng and the limit of quantification 0.66 ng.

The quantification of fully separated aflatoxin B1 was performed by comparison with aflatoxin B1 standard using formula and checking the linearity.


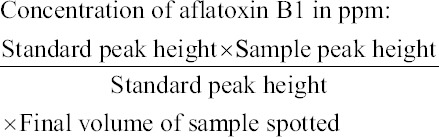


## Results and Discussion

The recovery percentage of aflatoxin B1 was 90% in the present study. The contamination of aflatoxin B1 was found to be 33% in livestock compound feed and 24% in feed ingredients in the present study, among the livestock compound feed analyzed for aflatoxin B1, except Pig adult and creeper mash, all other were within the safe limit of Indian standards (cattle feed = 50 ppb and pig and poultry feed = 20 ppb).

Among the 48 livestock compound feed samples analyzed (Table-1), 33% (16 of 48) were contaminated with aflatoxin B1 among them 26% (6 of 23) cattle feed samples shown the mean concentration of 32 ppb which was lower the Indian standards(50 ppb). In poultry feed, 35.2% incidence was found (6 of 17) at concentration of 13.4 ppb which was below the Indian standards (20 ppb). An incidence of 66.6% (2 of 3) was found in pig creeper mash with a mean concentration of 20 ppb and in pig adult mash an incidence of 50% (2 of 4) with a mean concentration of 30 ppb which is higher than the Indian standards for pig feed (20 ppb). Similar studies were conducted by Dhand *et al*.[8] in dairy cattle feeds and reported 75% of feed samples (21 of 28) were contaminated with aflatoxin B1. Our findings are in agreement with earlier reports, where aflatoxin B1 was found to be widely distributed in feed stuffs [9-11]. In a study by Sarathchandra and Muralimanohar [12], aflatoxin B1 levels ranged between 50 and 80 ppm in animal feed. Out of 59 samples of feed analyzed by HPTLC, 47 samples were positive for aflatoxin B1 representing 79.66% with a concentration of 25.53 ppb [13].

**Table-1:** Aflatoxin contamination in compound feed and feed ingredients.

Feed/feed ingredients	No. of samples analyzed	No. of positives	Incidence	Mean (in ppb)	Range (in ppb)
Compound feed	48	16	33.3	-	-
Cattle feed	24	6	26	32	20-60
Poultry feed	17	6	35.2	13.4	10-20
Pig creeper mash	3	2	66.6	20	20-20
Pig adult mash	4	2	50	30	30-30
Feed ingredients	49	13	24.5	-	-
Cotton seed cake	7	3	42.8	23.3	1040
Groundnut cake	5	3	60	23.3	20-30
Soyabean cake	3	1	33.3	50	-
Sunflower DOC	3	1	33.3	10	-
Maize	13	5	38.4	62	-
Wheat bran	2	0	0	-	-
Red gram	4	0	0	-	-
DO rice bran	4	0	0	-	-
Jowar	1	0	0	-	-
Feed by products	7	0	0	-	-
Grand total	97	29	30	-	-

For Feed ingredients (Table-1), 24.5% (13 of 49) shown contamination of aflatoxin B1 in cotton seed cake, groundnut cake, soyabean cake, Sunflower de oiled cake, wheat bran, maize, red gram de oiled rice bran, jowar and feed by products. An incidence of 42.8% (3 of 7) with mean concentration of 23.3 ppb of aflatoxin B1 was seen in Cotton seed cake. In groundnut cake, an incidence of 60% (3 of 5) was contaminated with mean concentration of 23.3 ppb. An incidence of 33.3% (1 of 3) soyabean cake was contaminated with aflatoxin B1 at a concentration of 50 ppb. In sunflower de oiled cake (1of 3) 33.3% found positive for aflatoxin B1 at concentration of 10 ppb. The highest concentration was seen in maize sample with mean concentration of 62 ppb showing incidence of 38.4 (5 of 13 samples). Maize, being the world’s important staple food [14] has been extensively studied for mycotoxins contamination as it has been found (among cereals) a very good substrate for fungal growth and toxigenesis[15], our results are in accordance with other worldwide surveys indicate the contamination of maize with aflatoxin Janardhana *et al*.[16], Sangare-Tigori *et al*. [17] andKhatoon *et al*. [18]. Whereas wheat bran, red gram, de oiled rice bran, jowar and feed by products are free from aflatoxin B1 in the present study. In a study, Mahammadi *et al*.[19] reported that among 152 samples of rice analyzed, 75% showed levels of aflatoxin B1 contamination with the mean of 0.671 ppb. Substantiating the present results, Anjum *et al*.[20] reported the overall incidence of 6% of aflatoxin B1 with average and maximum contamination levels of 37.62 and 56 ppb, respectively.

The results revealed low average aflatoxin concentration than the permissible levels, for livestock compound feed samples and feed ingredients except Pig creeper mash, pig adult mash, soyabean cake and maize. The results of the present study showed a higher incidence and contamination of aflatoxin B1 in pig creeper mash, pig adult mash, soyabean cake and maize, fluctuations in environmental temperature and humidity, rainy season and moderate temperature during preharvest period, intermittent showers during harvesting traditional harvesting practices and inadequate storage facilities induce fungal contamination and accumulation of mycotoxins [21]. Aflatoxin B1 levels in animal feed and feed stuff are important to human health since approximately 1-2% of the aflatoxin B1 in the animal feed is transformed to aflatoxin M1 in milk. Therefore, aflatoxin B1 concentration in feed above standards may result in milk containing a higher aflatoxin M1 [22]. In developing countries, many individuals are chronically exposed to low levels of aflatoxins in their diet [23].

## Conclusion

Our studies revealed that HPTLC assures good recovery, precision and linearity in the quantitative determination of aflatoxin B1 extracted from Livestock compound feed and feed ingredients. Our worked showed that 30% of livestock feed (33%) and feed ingredients (24%) were contaminated aflatoxin B1. There is a need for identifying the source of contamination, executing control measures, enabling better risk assessment techniques, and providing economic benefits.

## Authors’ Contributions

KK and MT have designed the plan of work. KK carried out the Laboratory work and analyzed the results. KK and TM drafted and RK revised the manuscript. All the authors read and approved the final manuscript.
